# The Oklo uranium reactor: a natural analogue informing preparedness in radionuclide-related man-made accidents

**DOI:** 10.3389/fpubh.2026.1972731

**Published:** 2026-09-29

**Authors:** Piero Andrea Ketura Ngouba Madinda, Tomoko Steen, Jean Paul Gonzalez

**Affiliations:** 1 Biohazardous Threat Agents & Emerging Infectious Diseases, Department of Microbiology and Immunology, Georgetown University, Washington, DC, United States; 2 Department of Microbiology and Immunology, School of Medicine, Georgetown University, Medical Center, Washington, DC, United States

**Keywords:** Chernobyl, Fukushima, Mounana, natural ionization, nuclear accidents, Oklo natural reactor, preparedness, radionuclides

## Abstract

**Introduction:**

The long-term health impact of radiation exposure is poorly documented, both in the context of accidental exposure and in that of natural exposure. While health authorities are mobilized to monitor health impacts in cases of accidental exposure—albeit often for a limited duration—the populations concerned by long-term natural exposure receive very little, if any, follow-up. Our research hypothesis focused on the health policies implemented during these events, and specifically on whether observing their health impact—as well as studying and monitoring natural radiation exposure within the affected populations—could provide useful guidance for the health officials responsible for managing such events.

**Methods:**

To this end, we conducted an exhaustive literature review including bibliographic data base, “grey literature” (internet sources and interviews) to gain the most comprehensive understanding possible of how these events are managed and their consequences for health.

**Results:**

Oklo demonstrated that natural geological containment could immobilize radionuclides over extremely long periods with minimal environmental impact. In contrast, Chernobyl, Fukushima, and Mounana showed that recovery outcomes depend heavily on containment design, long-term monitoring, and governance. The findings highlight the need for stronger international regulations, continuous environmental surveillance, and improved accountability for radionuclide-related activities.

**Discussion:**

The Mounana uranium mining incident in Gabon demonstrates how inadequate containment, poor worker protection, and insufficient environmental monitoring can lead to long-term human and ecological consequences from radionuclide exposure. In contrast, the Oklo natural reactor highlights the value of natural analogue systems for improving containment strategies, regulatory oversight, long-term surveillance, and preparedness for anthropogenic nuclear and mining-related disasters.

## Introduction

On a global scale, 80% (~2.4 mSv) of radiation exposure comes from naturally occurring radioactive elements, yet the public predominantly thinks it comes only from man-made disasters (1). Radiation has been part of the world for billions of years before humanity. Either natural or anthropogenic, radiation effect exists in two forms: non-ionizing and ionizing. Non-ionizing radiation such as radio waves and light lies on the lower energy end of the electromagnetic spectrum, below ionizing radiation such as X-rays, gamma rays, alpha and beta particles. In contrast to non-ionizing radiation, ionizing radiation can damage DNA and cause cell injuries, leading to Acute Radiation Syndrome (ARS) at high-dose exposure and carcinogenesis at lower doses over time (2). As a result, ionizing radiation represents a significant threat to public (radiation sickness, cancers, genetic mutations, reproductive disorders) and environmental health (Radionuclides caried by air, water, and soil contaminate ecosystems, entering the food chain through plants, animals, and aquatic organisms). Man-made ionizing radiation, on the other hand, brought forth tremendous medical, electrical, and nuclear advancements.

Over the past few decades, several anthropogenic radiation-related disasters have occurred, causing significant damage with long-lasting effects. Some of these high-impact disasters include Chernobyl (Soviet Ukraine), Fukushima (Japan), and Mounana (Gabon).

While these long-standing effects are inevitable, nature provides exceptional evidence of the successful long-term containment of nuclear waste through the Oklo natural nuclear fission reactor in Gabon, Africa. The Oklo site demonstrate that effective containment of radionuclides leads to minimal environmental impact significantly decreasing the need for recovery. Natural analogues, such as OKLO, necessitate to be integrated within disaster preparedness, monitoring, and governance policies. This review examines the Oklo natural nuclear reactor, compares its effects with those of man-made nuclear disasters, and evaluates the implications for regulation efforts.

## Materials and methods

All literature source sand data were identified through targeted searches of websites and institutional databases (PubMed, Scopus, Web of Science, Google search, etc.) using predefined keywords (e.g., radiation exposure, natural radiation, ionizing radiation, uranium, radon, cancer, geographies of natural ionizing zones). All sources, including grey literature, were evaluated for their origin (i.e., institute, agency, individual), relevance, transparency, consistency with published data, and potential biases. Data from interviews were coded and analyzed according to the themes explored and their consistency with the know literature.

### Study cases

The Natural Nuclear Reactor of Oklo (Gabon). Nuclear reactors are usually associated with nuclear power plants, research facilities, and naval vessels, all of which are man-made. Nature demonstrated once again that it has a head start on human activity (3). In 1972, French researcher Francis Perrin and his colleagues discovered a natural nuclear reactor from uranium mining in the Republic of Gabon, Central Africa. Mostly located at Oklo (14 at Oklo, 1 at Okelobond, and 1 located 30 km southeast from Oklo, there is a total of 16 known separate zones in the uranium deposits that functioned as natural fission reactors (4). Studies reveal that the Oklo reactor began operating about 2 billion years ago for 0.6–1.5 million years and certainly stopped due to water depletion. In fact, ground water served as a moderator, cooling down the reactor similarly to anthropogenic reactors, allowing such chain reactions to persist over time. The nuclear waste generated by the fission reactions was contained deeply under the ore by granite, sandstone, and clay in the surrounding area (5). This finding confirms that radioactive decay has remained constant for billions of years within nature and that nuclear waste can be securely contained in the long term. Therefore, anthropogenic radionuclide-related engineering and monitoring systems must use Oklo as a model for improving containment strategies.

The Chernobyl accident (Soviet Ukraine) of 1986 refers to the explosion of a reactor at the town’s nuclear power plant, followed by a 10-day fire, which led to a large release of radionuclides across Soviet Ukraine and the rest of Europe (6). Immediate health effects of the incident resulted in 134 workers being ill from radiation exposure, including several cases of ARS. Moreover 28 individuals died within the first 3 months following the accident. Since the incident, over 6,000 cases of thyroid cancer have been documented among children and adolescents, with an increase in leukemia cases (7). Indeed, Radioactive iodine-131, accumulated in pastures, entered the local milk supply, and concentrated heavily in children’s thyroid glands (8).

Fukushima Daiichi Accident (Japan) of 2011 is a nuclear power plant-related incident. This accident occurred after a magnitude 9.0 earthquake, followed by multiple tsunamis that disabled the power supply and cooling systems for the reactors (9). There were no deaths directly tied to the radiation exposure, but rather to the stress put on the population with the massive evacuation (9–11). The land and the ocean were highly contaminated with radionuclides, affecting vegetables, fruits, and the marine ecosystem (12).

The Mounana uranium mining pollution (Gabon). The Mounana uranium mining site in Gabon has become a source of persistent radioactive pollution following decades of uranium extraction activities, spanning from 1958 to 1999. During this period, extraction operations were subject to little—or inadequate—oversight by the COMUF (Compagnie des Mines d’Uranium de Franceville) company. Consequently, tons of radioactive residues contaminated the environment, potentially exposing workers and local communities to chronic health risks due to inadequate containment and insufficient protective measures. This incident highlights the major challenges associated with environmental monitoring, governance, site rehabilitation, and the long-term management of industrial activities involving radionuclides.

## Results

Oklo and Man-made disasters Containments (Table 1). Oklo fission reactions had a natural strategy for radioactive waste containment. Geological immobilization within the uranite ore matrix prevented radionuclides from moving freely into the environment (5). Therefore, nuclear waste was naturally contained within the sedimentary rocks under Oklo, leading to long-term stability.

**Table 1:** Accidents related to the use and/or operation of nuclear products and their impact on health.

Feature	Chernobyl, USSR Ukraine (1986)	Fukushima Daiichi, Japan (2011)	Mounana, Gabon (1962–1999)
Incident type	Sudden nuclear reactor explosion and meltdown.	Sudden power failure and multi-reactor core meltdown.	Decades-long industrial uranium mining and waste mismanagement.
Primary cause	Flawed High-Power Channel-Type Reactor (RBMK reactor) design and operator errors.	9.1 magnitude earthquake and 14-m tsunami disabling cooling systems.	Neglected environmental safety standards, unventilated tunnels, and open dumping.
Containment structure	None. No robust physical containment building existed.	Yes. Secondary steel/concrete containment vessels, though damaged.	None. Open pits, tailing piles, and unfortified underground tunnels.
Major hazards released	High levels of short- and long-lived fission products of Iodine-131, and Cesium-137.	Low-to-moderate atmospheric release of Iodine-131 and Cesium-137.	Alpha radiation from inhaled uranium dust, Radon gas, and heavy metals in rivers.
Nuclear and radiological event scale (INES rate)	Level 7 (Major Accident)	Level 7 (Major Accident)	Unrated
Direct health impacts	28 acute deaths; thousands of pediatric thyroid cancers.	No acute deaths; 2,300 secondary deaths from chaotic evacuation.	High rates of respiratory illness and suspected lung cancers among miners (lack of clinical tracking).
Environmental impact	A 2,600 km^2^ permanent exclusion zone across Ukraine and Belarus.	A heavily monitored, gradually shrinking exclusion zone in Japan.	Radioactive tailings left in forests; Mitemba River contaminated above safety limits.

Chernobyl’s reactor lacked a full-pressure containment structure, relying on a steel-lined concrete dome that could trap radiation in the event of such an accident. As a result, a containment failure at the nuclear reactor led to the release of a large number of radionuclides into the environment, immediately affecting the surrounding ecosystem. Even though a sarcophagus was built to reduce further radiation exposure, the damage had already been done (8).

Fukushima’s containment was more efficient than Chernobyl’s. This outcome was due to the containment structure of the power plant, as well as the cause of the accident (13). The Daiichi reactors were protected with steel-lined concrete, which served as the primary containment strategy. The containment structure held largely until the cooling disruption and hydrogen buildup, leading to the meltdown of the three reactors (14). Consequently, radionuclide containment worked as expected until the loss of cooling due to the earthquake and tsunamis. Studies also found that the nuclear waste released into the environment was lower than that from Chernobyl.

Mounana ionization was poorly contained. In 1990, a dam was constructed across the Ngama-Boungou Creek to contain about 7 million tons of the remaining tailings (15). Over 2 million tons of tailings had already been released into the creek, contaminating the Mitembe River downstream before the dam’s construction (16, 17). According to experts, the dam was not considered adequate containment for the incident. Furthermore, leakage from the dam down the river system has been ongoing with no improved containment strategy nor environmental monitoring thus far. This lack of proper containment leads to chronic exposure to radionuclide residues. The Oklo reactor provides a geological analogue for long-term radionuclide immobilization, where nuclear waste remained largely trapped within sedimentary structures over a long period. On the other hand, anthropogenic designs such as those at Chernobyl and Fukushima show that containment effectiveness highly depends on the reactor design and the nature of the incident. Further, poorly managed industrial sites such as Mounana illustrate insufficient long-term governance. Consequently, containment affects radionuclide mobility in the environment, which scales exposure and recovery needs.

Oklo and Man-made disasters Recovery. Oklo facilitated the ecosystem’s adaptation and recovery through its natural containment structure, which prevented radionuclides from moving freely in the environment (5). Minimal environmental disruption was documented as the uranium underwent fission over hundreds of thousands of years. Its efficient self-containment strategy did not require large-scale remediation as nuclear waste did not spread into the environment.

Chernobyl’s initial failure of reactor containment led to greater radiation exposure that exacerbated recovery needs. The construction of the sarcophagus limited further damage, but initial waves of radionuclides had already spread widely across the former Soviet territory and the rest of Europe. This initial failure was enough to result in the loss of multiple lives, a large increase in cancer cases (thyroid), animal and plant genetic anomalies, and forest and water system pollution. The high level of environmental contamination required subsequent remediation planning to ensure the recovery of the surrounding ecosystems. Continuous monitoring of radiation levels, evacuation of the population, decontamination incentives, and an upgrade in reactor containment all played a crucial role in the recovery from the tragic accident. Even though the site itself is deserted by humans within a 30 km radius, it has become an animal sanctuary and one of the largest natural reservoirs despite radiation levels still considered high, demonstrating nature’s resilience and animal adaptation. Compared to Oklo, whose radionuclides did not spread into the environment, Chernobyl’s opposite outcome led to a high-impact recovery.

Due to containment strategy and circumstances, Fukushima resulted with a smaller atmospheric release of radioactivity compared to Chernobyl leading to moderate recovery needs (18). The Daiichi plant, with the U.S. Nuclear Regulatory Commission (NRC) recommended protection, limited damages resulting in moderate recovery needs. Contamination still occurred but engineered Advanced Liquid Processing Systems (ALPS) and decontamination measures help further reduce the risks. Long-term monitoring studies have not been able to link health effects, including cancer, from the incident to the Fukushima population (19). Such analyzes corroborate the moderate need for recovery compared to Chornobyl, while still falling short of Oklo’s successful containment. Continuous environmental monitoring and imposed limits on uranium and other isotopes in vegetables, fruits, and marine products have been ongoing, complementing recovery incentives.

Mounana’s recovery is more complicated to assess. Due to the cessation of occupational health services by the French company COMUF and their lack of transparency, the health effects among miners could not be traced, as many could not afford healthcare (15, 20). Although protagonists such as Jacqueline Godet shared their testimonies and gave voice to workers through the Mounana Association, the lack of epidemiological data prevented a complete assessment of the damage inflicted on workers, their relatives, and the population still living in the area. The lack of long-term environmental and population monitoring, along with governance failures, creates uncertainty and impedes recovery pathways in Mounana.

Consequently, successful containment is the secure path for recovery, or even better, to prevent the need for recovery based on nature’s model and observed outcomes from man-made disasters. The natural reactor encourages progress on mankind’s learning curve regarding engineered control and monitoring incentives, leading to improvements in radionuclide-related activity regulations.

Implications for Regulations. Natural analogue-informed technical regulation Oklo reactor provides a natural model for long-term nuclear waste containment. It must also be integrated into geological risk assessment to help inform engineering solutions and emergency response strategies for radionuclide-related structures such as Nuclear Power plants and uranium mines. It should also be used in repository design and long-term containment modeling. Furthermore, the fissile use of groundwater as a moderator in nuclear reactions can inform understanding of the moderation mechanism, as it typically uses surface water such as lakes, rivers, and oceans. Oklo can thus inform engineering modeling systems through certain natural conditions, even if not replicated. Consequently, overlooking natural analogues such as Oklo is a missed opportunity to improve technical regulation and the efficiency of nuclear waste management and related disaster response.

Standardization of Disaster Preparedness and Monitoring. Chornobyl and Fukushima teach us a lot regarding preparedness and monitoring. The need for a sarcophagus at Chernobyl demonstrated the importance of preparedness, specifically initial containment. In fact, Oklo’s ability to effectively prevent waste from spreading supports the idea that proper initial containment structures can avoid a situation of aftermath in the first place. Furthermore, both Chernobyl and Fukushima have been conducting environmental monitoring and surveillance to assess radiation risks and reduce exposure since the incidents. Such consistency in environmental monitoring and surveillance was made possible through the continuous efforts of national systems and the support of international scientists.

It is important to note that regulations regarding containment strategies already exist and are upheld by international organizations such as the IAEA. However, compliance is uneven due to the lack of enforcement power. A way to enforce those containment standards to avoid new incidents like Chernobyl is for regulatory frameworks to require participation in international peer-review mechanisms. This approach will incentivize engagement across all countries, improve containment strategies upon inspections, helping to prevent and mitigate disasters. This approach, which must be extended across all countries, lays firm ground for the enforcement of monitoring regulations. Chernobyl and Fukushima provided strong examples of continuous monitoring since the incidents occurred. Therefore, long-term environmental monitoring and community follow-up must be integrated and standardized across countries, especially after disaster occurrence. This incentive should last for decades through the concerted efforts of national systems and international scientists.

Additionally, to containment inspection and monitoring enforcement, stress-testing regulations should be integrated on a global scale. The national systems in Ukraine proposed a competition among international scientists to replace the originally built sarcophagus. This competition triggered scientists to think outside of the box and generate the best proposal leading to the New Safe Confinement (NSC). Following this approach, proactive design improvement through scenario-based planning should be incorporated. Scientists worldwide would propose solutions to improve containment strategies for low-probability scenarios such as tsunamis.

Governance and accountability. The coexistence of one of the world’s greatest examples of radionuclide containment with one of the most under-examined mining-related radioactive contamination in Gabonese territory provides an instructive policy contrast. Companies such as COmpagnie GEnérale des MAtières nucléaires (COGEMA alias Orano Cycle) are aware of environmental and mining policies but hold a conflict of interest. Global South countries like Gabon have national mining and environmental regulations but lack the technical equipment to properly monitor radionuclide activity. As a result, incidents such as Mounana arise. Even though the company has ceased operations, the southeast town is still silently coping with the biological, psychological, and environmental effects. The incident demonstrates the need for an upgraded containment strategy and long-term environmental and community monitoring. Consequently, both governmental and international bodies need to enforce regulations governing radionuclides. Entities such as the International IAEA have a global impact on standardized radiation-related regulations but lack enforcement power, making compliance optional in most cases. Enforcement weaknesses also affect corporate accountability and post-closure responsibility, as witnessed in Mounana.

A comprehensive approach would include training local scientists and establishing long-term oversight funding. The IAEA’s work framework includes inspections, training capacity, and monitoring recommendations. The requirement to participate in international peer-reviewed mechanisms, as mentioned in the previous sections, will incentivize countries like Gabon to comply with international recommendations. This initial partnership will evolve into training national scientists to build scientific capability for long-term monitoring. In some cases, countries may not have enough scientists to train or a lack of monitoring equipment, hence the need for long-term funding. The creation of an international “radionuclide-related remediation fund” will support long-term monitoring capacity, especially in the global south. It will help provide equipment and support for long-term monitoring by international bodies such as the International Atomic Energy Agency (IAEA) in countries lacking scientific capacity. The oversight of those funds must be done by international bodies, including the IAEA, that have been working toward standardization of safety guidelines regarding radionuclide-related activities.

## Discussion

We acknowledge that the Oklo uranium reactor has limitations for assessing the risks of exposure to radionuclides including: Oklo’s very specific geological and environmental conditions, which differ significantly from the reactor’s initial conditions (groundwater chemistry, temperature, hydrology, and radionuclides); Oklo’s lack of insight into short-term accident processes, unlike Mounana and its extractive activities; the radionuclide inventory at Oklo is not necessarily being representative of nuclear accidents. Ultimately, Oklo should be considered a complementary natural analogue and reinterpreted considering contemporary direct models for predicting or managing radioactive accidents.

Instead of nuclear power plants, the Mounana (Gabon) incident involves uranium mines. The French nuclear company COGEMA, conducted uranium mining in the southeast of the country from 1960 to 1999, through its subsidiary COMUF. Mining sites that also included the Oklo mine, which hosted the very same uranium ore that French scientists later discovered was a natural nuclear reactor (21). Studies have shown that the company dumped over 7 million tons of radioactive tailings into the Ngama-Boungou creek and surrounding ecosystems, contaminating the Mitembe river and affecting the lives of local communities (15). Workers also lacked appropriate PPE and were exposed to 5 times the international standard of 5 mSv (25). Whereas hospital officials claimed there were no mining-related cases, testimonies, such as that of the Mouna association for miners’ president, Jacqueline Godet, suggest broader health impacts among the population (22). Whether caused accidentally or intentionally, disasters linked to man-made radionuclides can result in long-lasting psychological, pathological, and environmental damage. Such incidents call for continuous improvement in remediation incentives to efficiently address the aftermath of anthropogenic disasters. Using a natural analogue, such as Oklo, as a comparative can pinpoint weaknesses in anthropogenic design.

## Conclusion

Ultimately, natural analogues like the Oklo reactor are underused but valuable for modeling long-term radionuclide containment to inform mitigation regulations of anthropogenic disasters. Natural systems and anthropogenic design have already demonstrated that containment determines the outcomes of accidents such as Chernobyl and Fukushima and can even prevent the need for recovery.

The undeniable contrast between Oklo natural mine and Mounana uranium mining unravels a strong need for policy improvement concerning containment inspections, environmental surveillance, and community monitoring. Finally, the Oklo region, in southeastern Gabon, located on the Central African basement, like the Katanga region in the Democratic Republic of Congo, also constitutes a uranium-rich environment, both natural and anthropogenic. In Oklo, limited research has focused primarily on environmental radioactivity, uranium migration, contamination, and exposure pathways, with limited assessments of cancer risks. In Katanga, particularly around the Shinkolobwe (the historical source of high-grade uranium ore used in the Manhattan Project and the 1945 atomic bombs) deposit and in the surrounding mining areas, studies have highlighted elevated gamma radiation, uranium contamination, and increased uric acid levels in local populations. These two environments differ from the Chernobyl and Fukushima nuclear accidents, which involved acute releases of radionuclides and significant environmental contamination with high levels of exposure (radiation-induced thyroid cancer in the case of Chernobyl). While these two sites are models of acute radioactive exposure, Oklo and Katanga offer complementary models of prolonged and localized exposure. All provide a comparative framework for studying the biological and epidemiological consequences of exposure to ionizing radiation, despite limited direct population-level cancer data for Oklo and Katanga. A comprehensive approach in regulation implementation will enforce standardization of containment strategies, long-lasting environmental and biological monitoring, and proactive design improvement to strengthen readiness. Finally, the integration of Oklo as a model for radionuclides containment by regulatory frameworks such as IAEA, must be conjugated with strong governance oversight to enforce policies that will mitigate and prevent radionuclide-related disasters.

## Data Availability

Publicly available datasets were analyzed in this study. This data can be found here: All sources of data are in the references directly accessible.
